# Advanced Extubation Strategy in a Pediatric Difficult Airway: Successful Use of a Cook Staged Extubation Set

**DOI:** 10.7759/cureus.104314

**Published:** 2026-02-26

**Authors:** Catarina Amarante Dias, Catarina C Chaves, Helder Pereira, Ana Martins Lopes, Patrícia Santos

**Affiliations:** 1 Anesthesiology, Unidade Local de Saúde da Região de Aveiro, Aveiro, PRT; 2 Anesthesiology, Unidade Local de Saúde de São João, Porto, PRT; 3 Surgery and Physiology, Faculty of Medicine, University of Porto, Porto, PRT

**Keywords:** airway management, bleomycin therapy, cook staged extubation set, difficult extubation, pediatric airway

## Abstract

We report the case of a 14-year-old girl with a large cervical venous malformation extending to the pharyngeal mucosa who underwent sclerotherapy with bleomycin and polidocanol. Given the risk of airway obstruction and bleeding, awake fiberoptic intubation was performed under sedation with ketamine and dexmedetomidine. Extubation was deferred until postoperative day 6 due to persistent edema and residual malformation. A staged extubation strategy using a Cook Staged Extubation Set (CSES) (Cook Medical, Bloomington, IN, USA) was selected to ensure safe airway control. The device was well tolerated for 12 hours without complications or the need for reintubation. To our knowledge, this is the first reported pediatric case describing the successful use of a CSES for planned extubation of an anatomically difficult airway. This case underscores the importance of individualized extubation planning and multidisciplinary collaboration in pediatric airway management.

## Introduction

The pediatric airway management literature predominantly focuses on intubation, with relatively limited discussion of extubation strategies in terms of both scope and scale [1]. However, data from the APRICOT study suggest a higher likelihood of complications following extubation compared with intubation [2]. One study examining tracheal extubation in pediatric patients with difficult airways reported an extubation failure rate of 5%, with severe consequences, including death [3]. We present a clinical case involving an anatomically difficult pediatric airway in a child with a large cervical venous malformation scheduled for sclerotherapy. The presence of the mass rendered the use of a laryngoscopy blade hazardous due to the high risk of bleeding and potential loss of airway control. To maintain safe access to the airway following the procedure, a Cook Staged Extubation Set (CSES) (Cook Medical, Bloomington, IN, USA) was employed. This device allows a guidewire to remain in the trachea after extubation, providing a conduit for rapid endotracheal tube reinsertion if required, without the need for laryngoscopy.

## Case presentation

**Figure 1:**
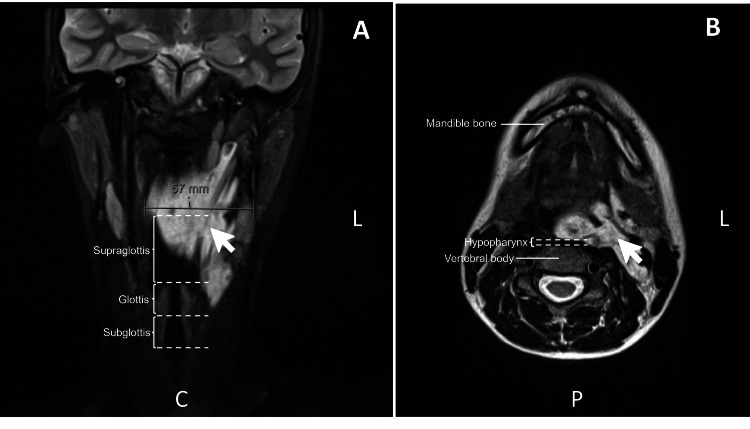
(A) Sagittal and (B) transverse magnetic resonance images of the contrasted cervical vascular malformation In the sagittal plane (A), the supraglottic (invaded by the venous malformation), glottic, and subglottic spaces are visible. In the transverse plane (B), the hypopharynx appears compressed by the mass, and the image also illustrates how a laryngoscope blade would come into contact with the lesion if used. The white arrow represents the venous malformation, and the dashed lines represent the limits of the supraglottic, glottic, and subglottic spaces in panel A and hypopharynx in panel B. C: caudal, L: left, P: posterior.

A 14-year-old girl (44 kg) with a large cervical venous malformation (Figure 1) was scheduled for sclerotherapy. The lesion extended toward the pharyngeal mucosa, resulting in oropharyngeal compression and deviation. She reported intermittent bleeding during eating, with no other airway stigmata. Given the anatomical location of the mass and the presence of bleeding, airway management techniques involving direct laryngoscopy or supraglottic airway devices were considered unsafe due to the high risk of severe hemorrhage and potential loss of airway control.

After discussion among the airway team, the patient, and her family, an awake tracheal intubation was planned. Fiberoptic-guided intubation was performed using a size 6 cuffed endotracheal tube under sedation with ketamine and dexmedetomidine, with topical anesthesia using lidocaine. Sedation was titrated to a University of Michigan Sedation Scale (UMSS) score of 2-3 [4], ensuring patient cooperation and maintenance of spontaneous ventilation. Intubation proceeded smoothly without coughing, desaturation, or hemodynamic instability.

The procedure itself was uneventful; however, due to the high risk of airway edema during the first 36 hours, the patient was transferred intubated to the pediatric intensive care unit.

On postoperative day 5, fibroscopic and ultrasonographic airway evaluation revealed significant edema, prompting initiation of corticosteroid therapy (dexamethasone 0.25 mg/kg twice daily). The following day, evaluation demonstrated resolution of edema, but residual malformation rendered laryngoscopy unsafe, and further bleomycin therapy carried a risk of rebound edema.

Given the patient’s cooperativeness and the potential difficulty of reintubation, extubation was performed using a CSES. The staged extubation wire was placed at the same length as the orotracheal tube, which was then removed. During this period, the patient was continuously monitored with pulse oximetry and electrocardiography and was clinically assessed for signs of respiratory distress.

Apart from hypersalivation, which resolved with atropine, no complications occurred. The wire remained in place for 12 hours, was well tolerated, and no ventilatory support was required. On postoperative day 9, the patient was safely transferred to the ward.

## Discussion

This report illustrates the potential of staged extubation techniques in selected pediatric difficult airway cases. While extubation conduits such as the CSES are recommended in adult difficult airway guidelines [5-8], their use in pediatric patients is rarely documented. To our knowledge, this represents the first published case describing the use of a CSES for planned extubation in a pediatric patient.

Rationale for a staged extubation

Extubation in the presence of residual anatomical abnormalities, edema, or procedural risk (e.g., after sclerotherapy or radiotherapy) carries a substantial risk of airway loss. For these patients, maintaining a conduit for rapid reintubation, such as a staged extubation wire, provides a critical safety margin [9]. In adults, staged extubation devices have demonstrated feasibility, high tolerability, and minimal adverse effects when used in selected patients [10]. However, evidence in pediatric populations remains largely anecdotal [11], reflecting a broader lack of structured extubation strategies for children with difficult airways.

Evidence landscape

Most pediatric extubation studies focus on predicting extubation failure and optimizing post-extubation ventilation rather than on strategic extubation planning. Weatherall et al. [1] recently emphasized the need for formal pediatric extubation algorithms that incorporate staged approaches and multidisciplinary planning. In a 2023 meta-analysis, Lu et al. [10] reviewed the use of the CSES in adults and children, finding the device safe and effective. Only two pediatric studies were included, both employing the Cook Airway Exchange Catheter (CAEC) rather than the full CSES [12]. Consequently, pediatric-specific data remain lacking. This has contributed to wide variability in pediatric airway guidelines regarding extubation catheters: some include them as a potential tool [13], though not all clarify their use in children [7], while others recommend against their use entirely [11].

Device tolerability and limitations

Our case demonstrated excellent tolerability of the CSES over a 12-hour period, consistent with findings in adult ICU patients [14]. However, tolerability beyond 24 hours, optimal guidewire dwell time, and the success and ease of reintubation using the CSES were not evaluated in this report. Additionally, our patient was cooperative and developmentally mature; therefore, this management approach may not be generalizable to younger or non-cooperative children.

Implications for practice

This case underscores the need for proactive extubation planning as an integral component of difficult airway management. In pediatric patients with high-risk anatomical abnormalities, a staged extubation protocol, supported by multidisciplinary discussion, imaging, and fiberoptic assessment, may enhance safety. Future pediatric airway guidelines should address the use of advanced extubation tools and provide clear criteria for their implementation, similar to the structured frameworks used by adult airway societies. We recommend that the use of such devices be limited to cooperative patients and confined to an intensive care setting, with continuous clinical and objective monitoring until the device can be safely removed.

## Conclusions

Extubation is a high-risk and often underemphasized phase of pediatric difficult airway management that warrants the same level of structured planning as tracheal intubation. This single-case report suggests that a staged extubation strategy using the CSES may be a safe, feasible, and well-tolerated option in carefully selected, cooperative pediatric patients at high risk of extubation failure. However, the absence of a reintubation attempt and the applicability of these findings primarily to developmentally mature, cooperative adolescents represent important limitations.

Maintaining an indwelling guidewire for potential rapid reintubation can provide an additional safety margin when residual anatomical distortion, airway edema, or procedure-related risk persists. The successful use of the CSES in this case supports consideration of its inclusion in pediatric difficult airway management pathways, as it may enhance extubation safety in carefully selected high-risk or borderline pediatric patients, provided that timing, monitoring, and technique are appropriately managed.
